# Influence of Biochar on Soil Nutrients and Associated Rhizobacterial Communities of Mountainous Apple Trees in Northern Loess Plateau China

**DOI:** 10.3390/microorganisms10102078

**Published:** 2022-10-20

**Authors:** Rafiq Ahmad, Jianen Gao, Zhe Gao, Abdullah Khan, Izhar Ali, Shah Fahad

**Affiliations:** 1 Institute of Soil and Water Conservation, Northwest A& F University, Yangling, Xianyang 712100, China; 2 Institute of Soil and Water Conservation, Chinese Academy of Sciences and Ministry of Water Resources, Yangling, Xianyang 712100, China; 3 College of Water Resources and Architectural Engineering, Northwest A& F University, Yangling, Xianyang 712100, China; 4 College of Agriculture, Guangxi University, Nanning 530004, China; 5 Department of Agronomy, Abdul Wali Khan University Mardan, Mardan 23200, KPK, Pakistan

**Keywords:** biochar, soil nutrients, soil enzymes, bacterial community structure, apple trees, northern loess plateau

## Abstract

Biochar application can enhance soil health and alter soil bacterial community structure. However, knowledge relating to biochar on soil nutrients of mountainous apple orchards and then assessing its effect on soil health, especially on soil microorganisms, is still scanty. Therefore, we evaluated the responses of six biochar treatments [Ck (0), T1 (2), T2 (4), T3 (6), T4 (8), and T5 (10) Mg hm^−2^] with a basal dose of chemical fertilizer on the soil nutrients under potted apple trees across 3, 6, 9, and 12 months, and then investigated the responses of the rhizobacterial communities. Experimental findings demonstrated that: (i) Across the months, the biochar-applied treatment (T5) compared to the control significantly enhanced soil nutrients, including soil pH (2.12 to 2.29%), soil organic matter (35 to 40%), total nitrogen (59 to 65%), ammonium nitrogen (25 to 33%), nitrate nitrogen (163 to 169%), and the activities of urease (76 to 81%), alkaline phosphatase (30 to 33%), catalase (8.89 to 11.70%), and sucrase (23 to 29%). (ii) Compared to the control, the biochar-applied treatment (T5) had a more desirable relative abundance of the bacterial phylum Proteobacteria (35.47%), followed by Actinobacteria (8.59%), Firmicutes (5.74%), and Bacteroidota (2.77%). Similarly, the relative abundance of the bacterial genera in the T5 was *Sphingomonas* (8.23%) followed by *RB41* (3.81%), *Ellin6055* (3.42%), *Lachnospiracea* (1.61%), *Bacillus* (1.43%), *Kineosporia* (1.37%), *Massilia* (0.84%), and *Odoribacter* (0.34%) than the control. (iii) Among the alpha diversity, the biochar-applied treatment (T5) revealed the highest Chao1 (20%) and ACE (19.23%) indexes, while Shannon (1.63%) and Simpson (1.02%) had relatively lower indexes than the control. Furthermore, positive correlations were found between the soil nutrients and some of the abundant bacterial phyla. Overall, the findings of this research demonstrated that biochar application at 10 Mg hm^−2^ (T5) along with the required chemical fertilizer is beneficial to improve soil health and pave the way for sustainable production in apple orchards of the northern loess plateau.

## 1. Introduction

Apple trees are the most dominant fruit trees after citrus, grapes, and bananas around the world [1]. According to Zhao et al. [2] and Zhu et al. [3], China is the leading apple producer in the world, with a cultivable area of 2.41 million hectares producing 40.92 million tons of apples in 2015. In China, the arid and semi-arid regions of Shaanxi province have made great efforts to the apple industry in recent years. At present, apple orchards act as a pillar industry in the region’s economic development [4], accounting for a quarter of China’s apple yield and one-seventh of the world’s apple yield [5]. However, the region’s average apple yields are still lower than the leading apple-producing countries, primarily due to the lower soil organic matter (SOM) and total nitrogen (T.N) [1,6,7]. SOM and T.N are the two promising indicators for soil fertility, which not only provide nutrients but can also provide a suitable environment that is conductive to plant growth [8]. Similarly, Neilsen et al. [9] reported that orchards using chemical fertilizers increase soil fertility (SOM and T.N). However, over the past few decades of apple production, some soil types in China have been acidified due to the overuse of chemical fertilizers, which greatly affected soil fertility and beneficial microbial activity [9,10,11]. Therefore, assuring the mechanisms of soil nutrient availability and microbial activity in the rhizosphere is critical for plant survival and ecosystem stabilization, which is beneficial for the sustainable production of apple orchards.

Acting as the earth’s mutualistic symbiotic associations, soil microorganisms are the unseen engines primarily involved in many beneficial soil functions, including the release of nutrients and minerals, and showing resistance to plant diseases [12,13]. The apple orchard is a complex soil ecosystem that often harbors a rich microbial community [14]. A wide range of biotic and abiotic factors affect microbial diversity [14,15]. Among the microbial diversity, bacteria are the most diverse and key players on the planet [16], and it is estimated that a single gram of soil surface is occupied by more than 1,000,000 bacterial species [17,18]. Moreover, bacteria are dominantly beneficial in the soil environment, as they not only indicate soil quality and sustainability of the ecosystem [19], but can also affect the basic soil physiochemical properties [20]. Prior studies have demonstrated that compost amendment [21], compost and fumigation [22], and manure application [23] improved soil properties and microbial diversity. Furthermore, Chen et al. [24] indicated that shifts in soil bacterial communities with mulching practices improved the quality and productivity of apple orchards after five growing seasons. Currently, biochar has been shown to reduce soil erosion [25] and overuse of chemical fertilizer [26,27] without compromising agricultural production [28]. However, studies relating to biochar on soil chemical properties, enzymatic activities, and bacterial communities and compositions are scanty in the mountainous apple orchards of the northern loess plateau.

Biochar is an environmentally friendly black carbon derived from the pyrolysis of raw materials (manure, organic wastes, crop residues, and bioenergy crops) in oxygen-deficient conditions [28,29,30,31]. The abundant micropore structure of biochar, the aromatic structure, and the largest surface area adjust the soil physical aspects [32,33]. Furthermore, the porous structure of biochar sequesters carbon and improves soil health and productivity [31]. Meanwhile, it can also (i) provide a suitable habitat for bacteria to colonize, grow, and reproduce, (ii) provide C sources, mineral nutrients, and enzymatic activities, and (iii) change the basic soil physiochemical properties [34,35]. Furthermore, biochar coupled with chemical fertilizers is advantageous in strengthening soil permeability, retaining moisture, and the nutrients improve soil fertility [36]. While the sole application of biochar is incapable of providing abundant nutrients for crop growth and productivity [37,38], the best option for biochar is to combine it with organic or inorganic fertilizers to enhance the effectiveness of SOM. However, studies related to the biochar application with inorganic fertilizers on the soil physiochemical properties, enzymatic activities, and bacterial diversity were insufficient in apple orchards. Therefore, this study selected potted apple trees with a similar age and height for the experiment. The aims of this research were: (i) to examine the impact of biochar application in conjunction with inorganic fertilizers on soil properties and enzymatic activities; (ii) to study the response of bacterial community structure and composition to biochar application; and (iii) to characterize the effect of biochar application on the relationship between the soil bacterial community and soil environmental factors. This study gives a theoretical basis to realize soil quality enhancement and conservation policies that are conducive to enhancing and further promoting the quality and productivity of orchards.

## 2. Materials and Methods

### 2.1. Soil and Biochar

This study used apple orchard loess soil from the northern section of the loess plateau located in Fangta village, Ansai District (36°51′ N, 109°19′ E) of Shaanxi Province, China. The experimental site has typically hilly and gullied topography with a mean annual temperature and precipitation of 8.8 °C and 505.3 mm. The collected soil had an initial pH, soil organic carbon (SOC), total nitrogen (T.N), and total phosphorus (T.P) of 8.85, 3.67 g kg^−1^, 0.38 g kg^−1^, and 0.57 g kg^−1^, respectively, and was classified as a silt loam (20.18% sand, 63.90% silt, and 15.92% clay) according to the United State Department of Agriculture (USDA). Biochar was produced from clipped apple branches during oxygen-limited conditions at a pyrolysis temperature of 550 °C and was provided by the Shaanxi Yixing Technology Co., Ltd., Xi’an, China. Biochar had an initial pH, organic carbon, total nitrogen, total phosphorus, and total potassium of 9.52, 467.47 g kg^−1^, 4.55 g kg^−1^, 0.95 g kg^−1^, and 7.35 g kg^−1^, respectively. Biochar was ground and then sieved through a 2 mm sieve for the experiment.

### 2.2. Experimental Design

A pot experiment was conducted at the State Key Laboratory of Soil Erosion and Dryland Farming on the Loess Plateau, Institute of Soil and Water Conservation, Yangling, Shaanxi Province, China. The top and bottom diameters of the plastic pots were 30 cm and 20 cm, respectively, with a 30 cm height. Holes were made in the bottom of the pots for ventilation and drainage. Fifteen (15) kg of soil were added to each pot. The experiment consisted of three replications with six biochar treatments such as Ck (0), T1 (2), T2 (4), T3 (6), T4 (8), and T5 (10) Mg hm^−2^. A basal dose of 650 kg hm^−2^ urea, 120 kg hm^−2^ calcium superphosphate, and 310 kg hm^−2^ potassium sulfate were applied to all treatments. All the chemical fertilizers and biochar were thoroughly mixed in 0–20 cm of soil depth. Dwarf apple trees of similar age and height were planted in the pots. The pots were kept in the open natural environment and were watered with tap water in accordance to the climatic and growth conditions.

### 2.3. Sample Collection and Laboratory Analysis

A homogenized soil sample for chemical properties was collected from the plant rhizosphere of the designed pots at 3, 6, 9, and 12-month intervals. The collected soil samples across the months were air dried, sieved, and divided into two parts. The first part of the soil samples was analyzed for the soil pH, SOM, STN, and enzymatic activities including urease (UE), alkaline phosphatase (ALP), catalase (CAT), and sucrase (SC), and the second part was directly placed in a refrigerator at a temperature of −80 °C for the investigation of soil ammonium nitrogen (NH_4_^+^-N) and nitrate nitrogen (NO_3_^−^-N). Furthermore, the molecular analysis was only analyzed at the end of the experiment (12 months).

### 2.4. Soil pH, Organic C, and N Analysis

Soil pH was measured in a soil-to-water ratio (1:2.5) according to the method described by [16]. SOC was measured following the wet digestion of H_2_SO_4_-K_2_Cr_2_O_7_ in an oil bath at a temperature of 180 °C until the solution had boiled for 5 min to avoid incomplete oxidation [39]. Soil total nitrogen (STN) was evaluated by the K_2_SO_4_-CuSO_4_-Se distillation method (Semi-micro Kelvin technique), previously argued by Hua et al. [1].

### 2.5. Measurement of Soil Enzymes’ Activities

The soil enzymatic activities (urease, sucrase, alkaline phosphatase, and catalase) were analyzed using the procedure described by Guan et al. [40]. The UE activity was evaluated by the indophenol method; SC activity was evaluated by measuring glucose content following a 24 h incubation at 37 °C using sucrose as a substrate; soil ALP activity was assessed by the C_6_H_5_Na_2_O_4_P·xH_2_O method, and CAT activity was obtained from the KMnO_4_ titration method. All of the enzymatic activities were computed in milligrams per day (24 h) per gram.

### 2.6. DNA Extraction and Sequencing

Genomic DNA was extracted from 0.5 g of the mixed soil samples using the soil FastDNA^TM^ SPIN Kit (MP Biomedicals, Irvine, CA, USA) as described by the manufacturer. DNA concentration and purity were evaluated by a NanoDrop 2000 spectrophotometer (Thermo Scientific, Waltham, MA, USA) and later preserved at −20 °C for sequencing. The 16S rRNA V3-V4 region was sequenced for the bacterial communities with the forward primer 338F (5-ACTCCTACGGGAGGCAGCAG-3) and reverse primer 806R (5-GGACTACHVGGGTWTCTAAT-3) [41] using a thermal cycler machine Corbett (Table 1 and Table 2).

### 2.7. Processing of Illumina Sequencing Data

After PCR amplification, the extracted bacterial PCR products were purified using QIAquick PCR purification Kit (QIAGEN, Hilden, Germany). Then, the purified amplicons were pooled them in equimolar quantities, and sequenced them on an Illumina MiSeq Platform (Illumina, San Diego, CA, USA) [42]. Briefly, the high-quality sequences were assigned to OTUs at 97% identity threshold using UPARSE (http://drive5.com/uparse/, version 7.1, accessed on 30 May 2022) [43]. Annotation of each taxonomy was conducted with a standard confidence level (≥0.5) in the SILVA database [44].

### 2.8. Alpha and Beta Diversity Analysis

An OTU-based analytical technique was carried out to check species richness and evenness within a single microbiome of each sample. QIIME software (v1.8.0) (http://QIIME.org, accessed on 30 May 2022) was used for the estimation of the alpha diversity index (Chao1, ACE, Shannon, and Simpson indexes). The rarefaction curve and rank abundance curves were drawn (using the R software version 3.2) at a 97% identity threshold of the OTUs.

The similarity index of the community structure was determined by beta diversity at the OUT level of genotypes via weighted UniFrac distances and PCoA (principle coordinates analysis). The weighted UniFrac distance matrices were clustered and further estimated by the QIIME program (v1.8.0) (http://QIIME.org, accessed on 30 May 2022). They revealed phylogenetic relationships among the several communities and their abundance in the respective samples. PCoA revealed the similarity and dissimilarity matrix of the samples between the different treatments.

### 2.9. Statistical Analysis

Statistical analysis for soil environmental parameters and enzyme activities was assayed using computer-based SPSS16.0 software with the Duncan test (SPSS, Chicago, IL, USA). Alpha diversity, including Chao1, Shannon, Simpson, and ACE indices were computed using the QIIME program (v1.8.0) (http://QIIME.org, accessed on 30 May 2022). A Spearmen heatmap correlation analysis was carried out by using the R (3.2) program between the rhizobacterial abundance and soil physiochemical properties and enzyme activities. The dominant phyla were analyzed using the microbiome analyst, while the species richness and evenness of the rarefaction curves and abundance curves were made against the number of sequences [45]. Furthermore, the figures were drawn using Sigma Plot 14 software (Systat Software, San Jose, CA, USA).

## 3. Results

### 3.1. Soil Physiochemical Properties and Enzymatic Activities

Biochar application significantly improved soil physiochemical properties and enzymatic activities at the intervals of 3, 6, 9, and 12 months (Table 3). Compared to the control (Ck) treatment, the highest biochar application rate (T5) increased soil pH by 2.13, 2.12, 2.28, and 2.29%; SOM by 35, 37, 38, and 40%; T.N by 59, 60, 63, and 65%; NO_3_^−^-N by 164, 165, 163, and 169%; and NH_4_^+^-N by 25, 29, 30, and 33%. Similarly, enzymatic activities at the highest biochar addition rate (T5) optimized UE activity by 76, 78, 79, and 81%; ALP activity by 32, 30, 32, and 33%; SC activity by 23, 27, 28, and 29%; and CAT activity by 8.89, 9.89, 11.70, and 11.64%. In general, the results of the ANOVA revealed a significant (*p* < 0.05) impact of biochar application rates on the overall variables, with the exception of the lower biochar application rates (T1, T2), which demonstrated a similarity to the control (Ck) treatment.

### 3.2. Composition and Community Structure of Rhizobacterial Microbiome

**Figure 1:**
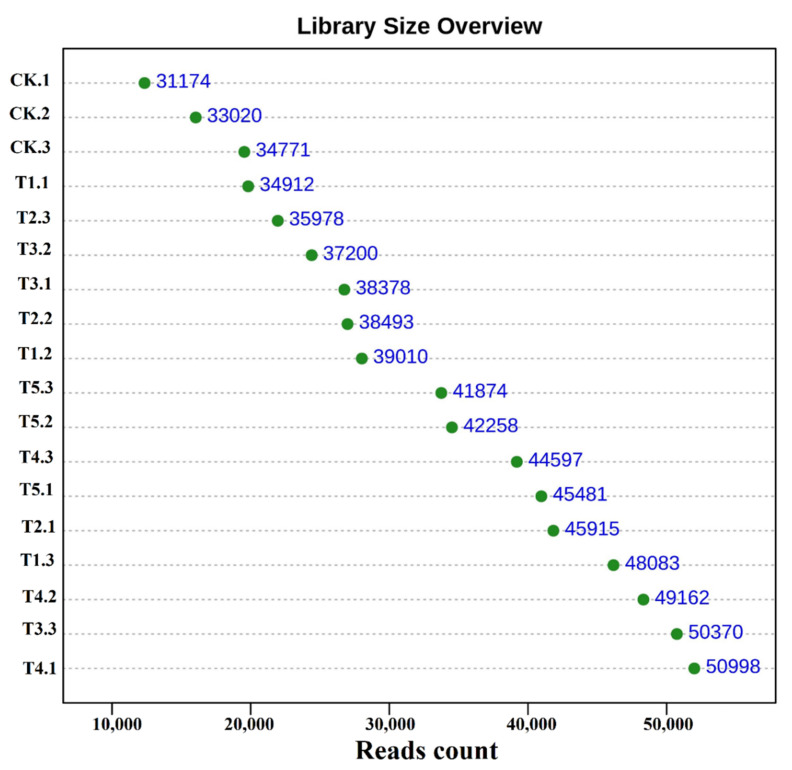
Library size overview of all the samples as influenced by various biochar application rates.

**Figure 2:**
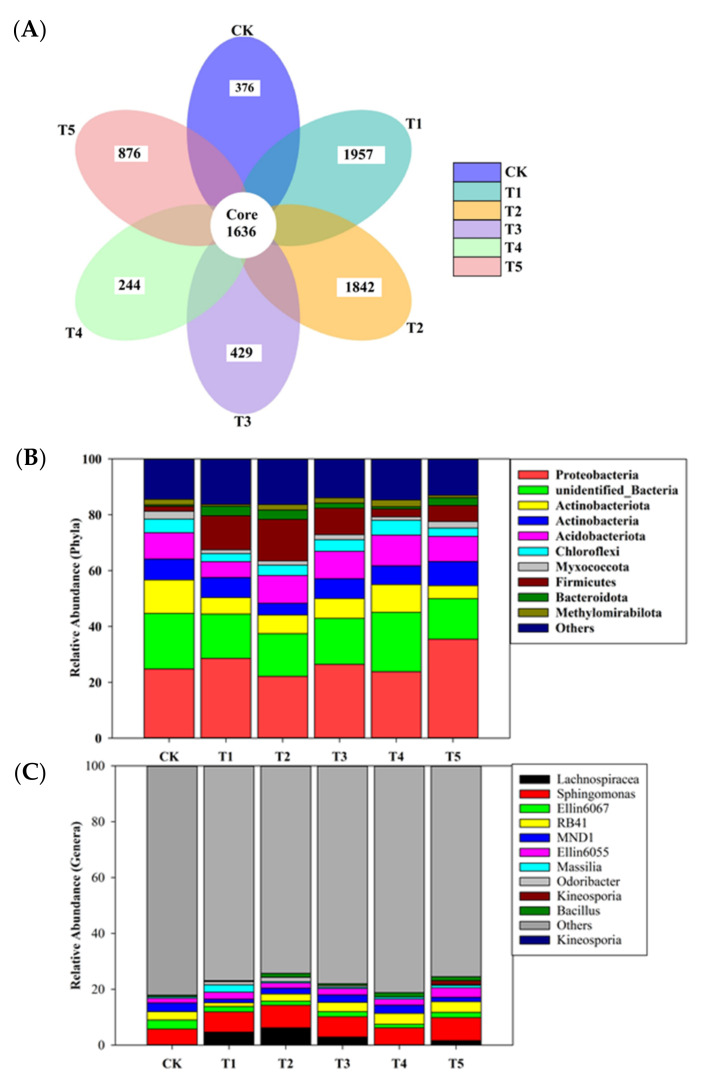
(**A**) Venn diagram illustrating number of unique and shared OTUs; (**B**) relative abundance of major bacterial phyla in each treatment; (**C**) relative abundance of major bacterial genera in each treatment.

After quality filtering of all the samples, a total of 741,674 reads of bacterial sequences with a mean of 41,204 ± 6247 per sample were obtained [min = 31,174 (Ck); max = 50,998 (T4)] (Figure 1). A total of 10,315 operational taxonomic units (OTUs) were obtained from the rhizobacterial soil of different treatments, in which all the treatments shared 1636 OTUs. All biochar-applied treatments demonstrated higher numbers of OTUs (ranged from 429 to 1957) than the control (376), with the exception of T4 (Figure 2A). Across the treatments, the highest relative abundance of the bacterial phylum in the T5 was Proteobacteria (35.47%), followed by Actinobacteria (8.59%), Firmicutes (5.74%), and Bacteroidota (2.77%). However, Acidobacteriota (8.99%), Actinobacteriota (4.66%), Chloroflexi (3.05%), Myxococcota (2.29%), and Methylomirabilota (0.88%) were the only phyla members in the T5 decreased by 4.35, 61.1, 37.5, 16.8, and 55.6%, respectively, compared to the control (Figure 2B). Similarly, the highest relative abundance of major genera in the T5 was *Sphingomonas* (8.23%), followed by *RB41* (3.81%), *Ellin6055* (3.42%), *Lachnospiracea* (1.61%), *Bacillus* (1.43%), *Kineosporia* (1.37%), *Massilia* (0.84%), and *Odoribacter* (0.34%) than the control (Figure 2C). However, *Ellin6067* (1.89%) and *MND1* (1.53%) were the only two generas decreased by 42.06 and 51.71%, as compared to the control.

### 3.3. Diversity and Species Richness of Rhizobacterial Microbiome

**Figure 3:**
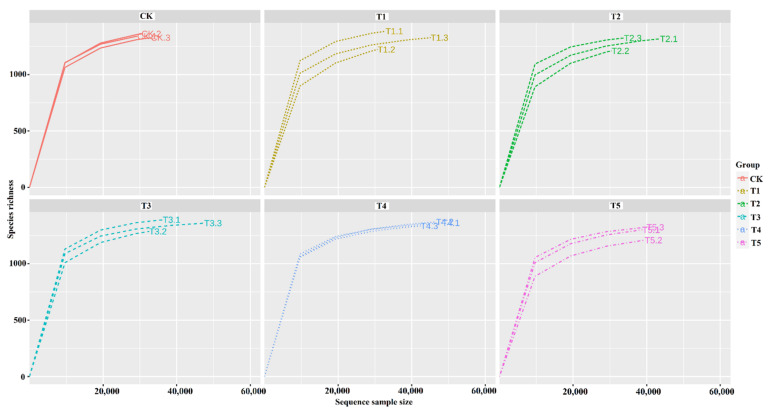
The rarefaction curve of bacterial species richness and sequencing sample size.

**Figure 4:**
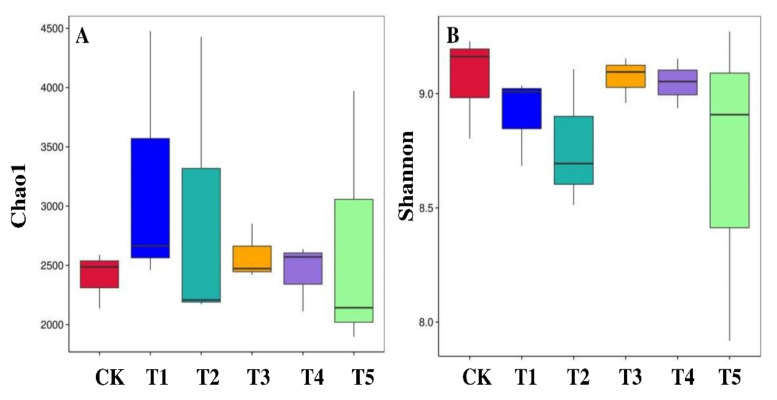
Bacterial alpha diversity measurements represented by (**A**) Chao1 index as richness and (**B**) Shannon index as diversity in each treatment, derived from the QIIME (http://qiime.org/, accessed on 30 May 2022) command α rarefaction.

Across the sample replicates, the rarefaction curve displayed higher sequencing depth and greater diversity. However, a closer association among the replicates of the same treatment was only observed in the Ck and T4 treatments. The abundance curve showed both species’ richness and evenness across the 18 samples (Figure 3 and Figure S1A,B). From the diversity analysis, treatment combinations significantly affected the diversity and abundance of rhizobacterial species. These indices were separately measured for each sample. Significant differential OTU richness computed by Chao1 (Figure 4A) and bacterial diversity computed by the Shannon index (Figure 4B) were observed in the rhizosphere of all the treatments. Among all treatments, Ck showed the highest bacterial diversity (Shannon index: 9.2) and the lowest OTU richness (Chao1: 2500), followed by T3 (Shannon index: 9.1) and OTU richness (Chao1: 2500). In addition, the ACE index was highest in T1 (ACE: 3800), followed by T2 and T5 versus in the Ck (Figure S2A). Whereas, the Simpson index showed no significant differences among the treatments; however, higher Simpson index (0.995) was found in the CK followed by T3, T4, and T5 treatments, respectively (Figure S2B).

### 3.4. The Similarity of the Rhizobacterial Microbiomes

**Figure 5:**
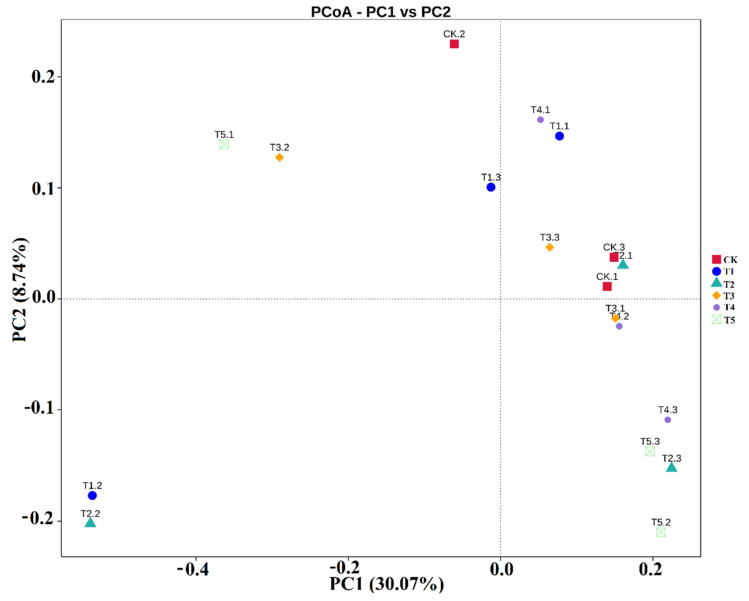
Analysis of beta diversity for estimating similarity or dissimilarity between the genotypes. PCoA (principal coordinate analysis) of weighted UniFrac distance.

The beta diversity in the rhizobacterial community of biochar-applied treatments was computed by the main coordinate components of PCoA, which revealed a clear tendency of the three replicates from the same treatment into the group together, except for one sample of T1 and T2, respectively, which was different from other treatments in terms of the rhizobacterial community. Furthermore, PCoA accounted for 38.81% of the total variation in the rhizosphere of the bacterial composition, 30.07% variation was explained by PCoA1 and 8.74% by PCoA2 (Figure 5). Similar consistent results were also found in the UniFrac-based hierarchical cluster analysis (Figure S3). The results demonstrated that all the samples were significantly clustered into different groups based on their taxonomic divergence, although not for each treatment. One sample of T1, T2, T3, and T5 clustered into one group, while all the remaining samples clustered into the second group.

### 3.5. Spearmen Correlation Analysis among Major Bacterial Phyla and Environmental Factors

**Figure 6:**
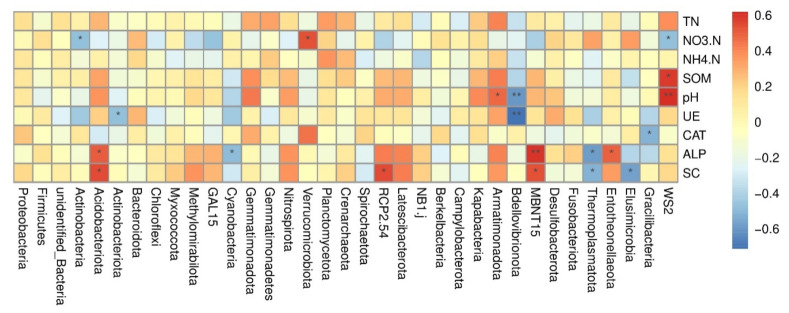
Spearmen heatmap correlation analysis of major bacterial phyla and environmental factors. The red mark indicates positive correlation, while the blue mark indicates negative correlation. The star marks indicate the significant level.

The Spearman’s heatmap indicated the relationship between bacterial diversity and soil traits (Figure 6). The analysis demonstrated that Acidobacteriota was positively and significantly correlated with SC and ALP. Actinobacteria and WS2 were negatively and significantly correlated with NO_3_^−^-N. WS2 was positively and significantly correlated with SOM and pH. All the other phyla have a low correlation with the environmental factors, but their effect was non-significant. Furthermore, the correlation analysis among the treatment combination and soil environmental factors was explored by the two main axes of dbRDA, which explained the 34.66% (dbRDA1) and 19.33% (dbRDA2) variation, respectively, out of the total variation of the data (Figure S4). The arrows indicate the magnitude of the correlation. The analysis indicated that soil pH, SOM, SC, and TN were more correlated with T3 treatment and also lie in the positive quadrant of the RDA axis. The CK treatment has no correlation with these environmental factors.

## 4. Discussion

Soil nutrients and fertilizer management significantly affect apple trees’ growth and production. Biochar amendment is continuously reported to improve soil health, plant growth, and yield in sustainable development. Furthermore, numerous studies argued that biochar application changed soil microbial abundance and community composition in different environments and rhizospheres [16,46,47]. However, studies relating to biochar blended with inorganic fertilizer on the soil nutrients (physiochemical properties and enzymatic activities), bacterial diversity, and their relationship under apple trees are still unclear. Soil bacterial diversity and community composition in response to biochar are important for the fertility of the orchards. As a vital factor for improving the fertility and soil health of mountainous apple orchards, this finding conducted the potted experiment with two-year dwarf apple trees to evaluate the response of soil nutrients and bacterial abundance and community composition to biochar application.

### 4.1. Soil Physio-Biochemical Properties

Ample evidence from scientific research reported that optimizing soil biogeochemical properties with biochar improves nutrient status and productivity [48]. However, limited water resources, poor soil structure, soil biodiversity, soil enzymes, and soil microbial biomass cycle processes directly or indirectly influenced soil properties [49]. In our findings, the optimization of soil properties (soil pH, SOM, T.N, NH_4_^+^-N, and NO_3_^−^-N) with the biochar application demonstrated consistency with the prior studies of [50,51], who suggested biochar as an ideal option for soil quality improvement and nutrient availability. Furthermore, Lehmann et al. [51] demonstrated that the larger surface area and numerous pores of biochar provide more space for microbial colonization. Similarly, Gul et al. [52] reported that the black color of biochar attracts more heat and thus may speed microbial growth and enzyme activity. Thus, the increment in the soil chemical properties in our findings corroborates the prior study of Gao et al. [53], who argued a positive relationship of biochar application with the soil pH, SOM, T.N, total organic carbon, and C:N ratio, which are known as the mechanisms for the improvement in soil fertility and thus lead to greater access for microbial colonization.

Soil enzymes are the essential biochemical processes in the soil environment [54,55]. However, changes in the soil enzymatic activities change the soil biochemical processes and microbiome aspects [56]. In this study, biochar application rates had significantly higher enzymatic activities than the control, demonstrating similarity with the earlier study [57]. A significant increase in the UE activity with biochar application (Table 3) demonstrated consistency with the prior study of Jindo et al. [58], who found a 40% increment with the biochar-blended compost. UE activity regulates nitrogen transformation in the soil, which is beneficial in nutrient cycling [59,60]. The increase in the ALP activity with biochar amendment (Table 3) is related to the improvement in the soil nutrients and corroborates previous studies [61,62,63]. However, the conflicting results of biochar on ALP have been attributed to differences in the biochar type, rates, production, and experimental conditions [64,65]. Furthermore, the highest CAT activity with biochar application in our study could be due to C cycling and microbial metabolism, as discussed by Khadem et al. [66]. Similarly, an increase in the SC activity with biochar was attributed to the biochar pyrolysis temperature (550 °C), and SOC became consistent with the latest study of Jiang et al. [67]. Overall, biochar application indicates a sign of positive impact on soil enzymatic activity, which reflects a valuable impact on nutrient cycling and soil biota.

### 4.2. Impacts of Biochar on Composition and Community Structure

Previously, Li et al. [68] and Hardy et al. [69] reported that biochar application alters the soil bacterial community composition. In the current study, biochar application demonstrated the highest relative abundance of Proteobacteria, Actinobacteria, Acidobacteriota, Chloroflexi, Firmicutes, and Bacteroidota than the control treatment. In terms of community composition and relative abundance, Proteobacteria occupied the largest portion of the soil, which is in line with the previous findings [70,71]. The possible explanation for the improvement in Proteobacteria abundance is that it is a eutrophic bacteria Fierer et al. [72], and the biochar application enhanced soil nutrients (Table 3), resulting in a higher Proteobacteria population. A similar finding by Ali et al. [16] demonstrated that adding biochar to paddy rice fields improved the abundance of Proteobacteria primarily due to an improvement in soil physiochemical properties. The biochar addition to soil improves Actinobacteria, which are important in the decomposition of SOM such as cellulose and chitin [16,73]. Furthermore, the increase in Firmicutes with the biochar addition can be classified as r-strategists [74], which may reduce the ecological risk posed by soil heavy metals, primarily due to the overuse of chemical fertilizers and pesticides in apple orchards. Consequently, the increase in the abundance of Bacteroidota with biochar application was attributed to the synergistic effects (co-metabolism or similar response patterns to the soil physiochemical and biological properties), as revealed by Ali et al. [16], Nielsen et al. [75], and Cottrell et al. [76].

The relative abundance of Myxococcota, Methylomirabilota, Actinobaceriota, Acidobacteriota, and Chloroflexi were higher in the control treatment and demonstrated a slightly decreasing trend with biochar addition rates (Figure 2B). In this study, the biochar application improved soil pH, resulting in a lower abundance of Actinobaceriota, Myxococcota, and Methylomirabilota. Similar to our findings, Ali et al. [16] and Yin et al. [71] reported the negative relationship of biochar application between the relative abundance of Actinobaceriota and soil pH in the rice field. Previous studies by Wei et al. [77] and He et al. [78] have demonstrated that Chloroflexi has a wide prospective for fixing carbon in poor soil nutrients. However, the reduction of Chloroflexi in this study is related to the increase in available nutrients (Table 3), which verifies the study of He et al. [78], who reported consistent results with the application of Rs-198 and the inoculated biochar. Biochar addition to soil decreased soil bacteria abundance up to 61% and their ratio with soil fungi was attributed to the fact that fungi were the primary decomposers of enhanced recalcitrant carbon from biochar and rice biomass [19]. In addition to a previous study, the higher soil pH caused by biochar can decrease with time (soil re-acidification), and the oxidation of biochar surfaces during ageing can also lower soil pH near biochar particles, resulting in less bacterial abundance [79].

In the case of the most abundant genera, *Sphingomonas*, *Lachnospiracea*, *RB41*, *Ellin6055*, *Kineosporia*, *Massilia*, and *Bacillus* were recorded in the biochar-applied treatments, in which the *Sphingomonas* genus belongs to the phylum Proteobacteria and plays a beneficial role in the availability of nitrogen to plants [80]. Huang et al. [35] argued that the *Lachnospiracea* genera in soil was positively associated with soil pH, which can be attributed to biochar application in our study. The decrease in the abundance of the genus *MND1* with biochar in our study could be attributed to adverse soil conditions that are less competitive in nutrient-rich and relatively healthy soil [81]. Similarly, a slight decrease in the *Ellin6067* genera with biochar could be related to the inhibition of soil nitrification [82], who found consistent results with the biogas slurry. In contrast to our results, *Ellin6067 and MND1* genera were slightly increased in biochar-treated pots after control. *Ellin6067* has been recognized as a putative ammonia-oxidizing bacterium [83,84], while *MND1* is capable of nitrification [85]. Overall, the findings demonstrated that the biochar-applied treatments were the most suitable habitat for beneficial bacteria in apple orchard soil.

### 4.3. Impact of Biochar on the Soil Bacterial Alpha and Beta Diversity

Alpha diversity describes the species diversity or species richness in an ecosystem, while beta diversity explains species diversity between two communities or ecosystems. Therefore, we determined both the alpha and beta diversity for bacteria under different applications of biochar (Figure 4, Figure 5 and Figure S2). The results demonstrated higher Chao1, Shannon index, and ACE index with biochar application; however, no significant differences were found in the Simpson index. Previously, biochar has been demonstrated to improve bacterial diversity indices, including NDMS and the Shannon index [86]. Similarly, an increase in Shannon and Simpson indices with the biochar addition was also previously reported by Ali et al. [16]. However, no significant differences have been found for the Shannon, Chao1, and Simpson indexes between the biochar and non-biochar treatments [87,88]. Furthermore, the beta diversity with biochar application promotes 30.07% and 8.74% of variation explained by PCoA1 and PCoA2, respectively (Figure 5), which was different from the study investigated by Ali et al. [16], who reported 51% (PCoA1) and 13% (PCoA2) of the variation in biochar application. Thus, we evaluated that adding biochar into the soil might have various effects on soil bacterial community composition, primarily due to various soil types, different plant rhizospheres, biochar types, and production conditions.

### 4.4. Correlation of Bacterial Communities and Environmental Factors

Soil physiochemical properties largely influence soil bacterial abundance and composition [80,89,90]. In this study, biochar application significantly improved soil nutrients (Table 3). Furthermore, the relationship of the Acidobacteriota was positively correlated with SC and ALP, while Actinobacteria and WS2 were negatively correlated with NO^−^_3_-N (Figure 6). In addition, the relationship of pH and SOM with WS2 was positive. A similar relationship between soil bacterial structure and composition with soil physiochemical properties was argued by [91]. Likewise, Ali et al. [16] and Zhang et al. [92] found a positive correlation of Proteobacteria, Acidobacteriota, and Chloroflexi with soil nutrients (pH, SOM, and T.N). Based on the above discussion, we investigated that biochar in conjunction with the chemical fertilizer provides a suitable condition for bacterial growth and enhances soil fertility, which could pave the way for sustainable production in the mountainous apple orchards of the northern loess plateau.

## 5. Conclusions

In this study, biochar in conjunction with chemical fertilizers improved the soil nutrients and increased the bacterial phyla and genera. Significant changes in the soil nutrients were observed in the T5 of the biochar-applied treatments. Similarly, the biochar-applied treatment (T5) altered the rhizobacterial microbiome and increased the desirable relative abundance of the bacterial phylum Proteobacteria, followed by Actinobacteria, Firmicutes, and Bacteroidota, while the relative abundance of the dominant genera in the T5 was *Sphingomonas*, followed by *RB41*, *Ellin6055*, *Lachnospiracea*, *Bacillus*, *Kineosporia*, *Massilia*, and *Odoribacter*. Furthermore, the biochar-applied treatment (T5), compared to the control, increased the bacterial Chao1 and ACE indices and decreased the Shannon and Simpson indices. The Spearman correlation of the soil nutrients was positively correlated with some of the most abundant bacterial phyla. Overall, the results of this study demonstrated that applying biochar at 10 Mg hm^−2^ (T5) along with the chemical fertilizers is beneficial to improve soil health and pave the way for sustainable production in the mountainous apple orchards of the northern loess plateau.

## Figures and Tables

**Table 1:** PCR reaction components system.

PCR Reaction System (50 µL)	Addition (µL)
2X Premix Taq	25
Forward primer (5 µM)	1
Reverse primer (5 µM)	1
DNA Template	3
Deionized distilled H_2_O	20

**Table 2:** Amplification programs of PCR reaction.

Stages	No. of Cycle	Temperature (°C)	Time (min)
Initial denaturation	1	94	5
Second denaturation	30	94	0.3
Annealing	1	52	0.3
Initial extension	1	72	0.3
Final extension	1	72	10

**Table 3:** Impact of biochar application on soil physiochemical properties and enzymatic activities across the months.

					3 Months				
Treat	Soil pH	SOM	STN	NO_3_^−^-N	NH_4_^+^-N	UR	CAT	ALP	SC
**Ck**	8.91 ± 0.06 ^a^	8.48 ± 0.19 ^a^	0.42 ± 0.05 ^a^	3.93 ± 0.15 ^a^	67.50 ± 3.90 ^a^	0.30 ± 0.03 ^a^	3.95 ± 0.09 ^a^	0.99 ± 0.05 ^a^	6.11 ± 0.35 ^a^
**T1**	8.93 ± 0.14 ^a^	8.75 ± 0.45 ^a^	0.53 ± 0.06 ^abc^	4.43 ± 0.82 ^a^	71.68 ± 2.39 ^a^	0.33 ± 0.05 ^a^	4.01 ± 0.07 ^ab^	1.09 ± 0.03 ^ab^	6.13 ± 0.40 ^a^
**T2**	8.98 ± 0.05 ^ab^	9.41 ± 0.30 ^b^	0.47 ± 0.07 ^ab^	6.43 ± 1.02 ^b^	78.69 ± 3.33 ^a^	0.51 ± 0.03 ^c^	4.09 ± 0.05 ^b^	1.12 ± 0.04 ^bc^	6.45 ± 0.16 ^ab^
**T3**	9.03 ± 0.02 ^ab^	10.30 ± 0.15 ^c^	0.55 ± 0.06 ^abc^	7.63 ± 1.04 ^b^	81.47 ± 5.20 ^b^	0.42 ± 0.06 ^b^	4.14 ± 0.06 ^bc^	1.19 ± 0.02 ^bc^	6.70 ± 1.18 ^ab^
**T4**	9.09 ± 0.02 ^b^	10.89 ± 0.22 ^d^	0.61 ± 0.13 ^bc^	9.43 ± 0.72 ^c^	83.82 ± 2.50 ^b^	0.45 ± 0.05 ^bc^	4.25 ± 0.04 ^c^	1.22 ± 0.09 ^cd^	7.11 ± 0.44 ^ab^
**T5**	9.11 ± 0.06 ^b^	11.52 ± 0.22 ^e^	0.67 ± 0.10 ^c^	10.42 ± 1.13 ^c^	84.77 ± 2.64 ^b^	0.53 ± 0.03 ^c^	4.30 ± 0.05 ^cd^	1.31 ± 0.06 ^d^	7.52 ± 0.27 ^b^
					**6 Months**				
**Ck**	8.95 ± 0.06 ^a^	8.56 ± 0.26 ^a^	0.46 ± 0.04 ^a^	3.98 ± 0.14 ^a^	68.20 ± 5.03 ^a^	0.32 ± 0.03 ^a^	3.94 ± 0.05 ^a^	1.02 ± 0.02 ^a^	6.15 ± 0.49 ^a^
**T1**	8.98 ± 0.06 ^ab^	8.78 ± 0.39 ^a^	0.52 ± 0.07 ^ab^	4.49 ± 0.20 ^a^	73.44 ± 8.14 ^a^	0.35 ± 0.07 ^ab^	4.05 ± 0.07 ^b^	1.12 ± 0.03 ^ab^	6.20 ± 0.52 ^a^
**T2**	9.02 ± 0.07 ^abc^	9.95 ± 0.35 ^b^	0.55 ± 0.07 ^abc^	6.60 ± 0.84 ^b^	81.95 ± 2.49 ^b^	0.54 ± 0.04 ^d^	4.09 ± 0.03 ^b^	1.17 ± 0.04 ^bc^	6.58 ± 0.22 ^ab^
**T3**	9.08 ± 0.04 ^bc^	10.62 ± 0.55 ^bc^	0.59 ± 0.05 ^bc^	7.77 ± 1.11 ^b^	84.04 ± 4.51 ^b^	0.43 ± 0.06 ^bc^	4.18 ± 0.06 ^c^	1.22 ± 0.03 ^bcd^	6.79 ± 1.13 ^ab^
**T4**	9.11 ± 0.04 ^c^	11.28 ± 0.17 ^cd^	0.67 ± 0.08 ^cd^	9.55 ± 0.58 ^c^	88.52 ± 1.50 ^b^	0.49 ± 0.06 ^cd^	4.27 ± 0.05 ^cd^	1.27 ± 0.10 ^cd^	7.28 ± 0.93 ^ab^
**T5**	9.14 ± 0.08 ^c^	11.69 ± 0.50 ^d^	0.74 ± 0.06 ^d^	10.58 ± 1.55 ^c^	88.57 ± 3.29 ^b^	0.57 ± 0.05 ^d^	4.32 ± 0.06 ^d^	1.33 ± 0.07 ^d^	7.85 ± 1.22 ^b^
					**9 Months**				
**Ck**	8.99 ± 0.01 ^a^	8.75 ± 0.33 ^a^	0.49 ± 0.05 ^a^	4.10 ± 0.33 ^a^	69.13 ± 14.07 ^a^	0.34 ± 0.04 ^a^	3.93 ± 0.09 ^a^	1.03 ± 0.06 ^a^	6.18 ± 1.11 ^a^
**T1**	9.04 ± 0.03 ^a^	8.86 ± 1.45 ^a^	0.57 ± 0.08 ^a^	4.53 ± 1.16 ^a^	74.79 ± 7.56 ^ab^	0.36 ± 0.03 ^a^	4.04 ± 0.07 ^ab^	1.13 ± 0.09 ^ab^	6.24 ± 0.39 ^a^
**T2**	9.06 ± 0.03 ^ab^	10.19 ± 0.28 ^b^	0.56 ± 0.07 ^a^	6.73 ± 0.92 ^b^	84.06 ± 4.54 ^bc^	0.60 ± 0.07 ^c^	4.13 ± 0.05 ^bc^	1.21 ± 0.03 ^bc^	6.67 ± 0.41 ^b^
**T3**	9.13 ± 0.01 ^bc^	11.56 ± 0.19 ^c^	0.62 ± 0.03 ^ab^	8.09 ± 1.68 ^bc^	85.12 ± 3.94 ^bc^	0.47 ± 0.05 ^b^	4.20 ± 0.06 ^cd^	1.27 ± 0.04 ^bc^	6.82 ± 1.06 ^b^
**T4**	9.16 ± 0.09 ^bc^	11.68 ± 0.40 ^c^	0.71 ± 0.09 ^bc^	9.78 ± 1.22 ^cd^	89.66 ± 2.81 ^c^	0.52 ± 0.06 ^bc^	4.31 ± 0.07 ^de^	1.32 ± 0.14 ^c^	7.43 ± 2.03 ^bc^
**T5**	9.19 ± 0.08 ^c^	12.03 ± 0.57 ^c^	0.81 ± 0.07 ^c^	10.82 ± 0.69 ^d^	90.30 ± 4.82 ^c^	0.61 ± 0.04 ^c^	4.39 ± 0.09 ^e^	1.35 ± 0.08 ^c^	7.97 ± 0.87 ^c^
					**12 Months**				
**Ck**	9.01 ± 0.01 ^a^	8.82 ± 0.21 ^a^	0.53 ± 0.06 ^a^	4.14 ± 1.82 ^a^	69.81 ± 30.94 ^a^	0.35 ± 0.05 ^a^	3.95 ± 0.11 ^a^	1.06 ± 0.03 ^a^	6.20 ± 1.37 ^a^
**T1**	9.08 ± 0.04 ^ab^	8.92 ± 1.52 ^a^	0.59 ± 0.16 ^ab^	4.64 ± 2.66 ^a^	76.16 ± 9.01 ^a^	0.38 ± 0.05 ^a^	4.08 ± 0.12 ^ab^	1.19 ± 0.28 ^a^	6.33 ± 1.35 ^a^
**T2**	9.10 ± 0.05 ^abc^	10.47 ± 0.56 ^b^	0.59 ± 0.09 ^ab^	7.32 ± 3.10 ^ab^	86.56 ± 5.01 ^b^	0.63 ± 0.06 ^d^	4.15 ± 0.11 ^abc^	1.33 ± 0.09 ^b^	6.83 ± 0.25 ^b^
**T3**	9.16 ± 0.01 ^bc^	11.65 ± 0.27 ^bc^	0.65 ± 0.07 ^ab^	8.52 ± 5.20 ^ab^	87.15 ± 8.11 ^b^	0.51 ± 0.04 ^b^	4.24 ± 0.11 ^bcd^	1.31 ± 0.09 ^b^	6.90 ± 1.16 ^b^
**T4**	9.19 ± 0.12 ^bc^	12.09 ± 0.27 ^c^	0.73 ± 0.12 ^bc^	10.13 ± 3.41 ^ab^	91.11 ± 4.58 ^b^	0.58 ± 0.10 ^bc^	4.33 ± 0.09 ^cd^	1.35 ± 0.13 ^b^	7.58 ± 0.94 ^c^
**T5**	9.22 ± 0.09 ^c^	12.38 ± 0.15 ^c^	0.87 ± 0.08 ^c^	11.17 ± 2.85 ^b^	92.97 ± 5.94 ^b^	0.65 ± 0.04 ^d^	4.40 ± 0.08 ^d^	1.41 ± 0.31 ^bc^	8.04 ± 1.41 ^cd^

Ck: control; T1: (2 Mg hm^−2^); T2: (4 Mg hm^−2^); T3: (6 Mg hm^−2^); T4: (8 Mg hm^−2^); T5: (10 Mg hm^−2^); SOM (Soil organic matter g kg^−1^); STN (Soil total nitrogen g kg^−1^); NH_4_^+^-N (Ammonium nitrogen mg kg^−1^); NO_3_^−^-N (Nitrate nitrogen mg kg^−1^); UR (Urease mg d^−1^g^−1^); CAT (Catalase mg d^−1^g^−1^); ALP (Alkaline phosphatase mg d^−1^g^−1^); SC (Sucrase mg d^−1^g^−1^). Different letters within the same column denote significant differences (*p* < 0.05) between the treatments.

## Data Availability

Data will be available on demand.

## Supplementary Materials

The following supporting information can be downloaded at: https://www.mdpi.com/article/10.3390/microorganisms10102078/s1, Figure. S1: (A) Observed species numbers (B) Rank abundance curve in the treatments with and without biochar, different color line represents different treatments. Figure. S2: (A) ACE and (B) Simpson index of OTUs for soil samples where biochar was applied. Figure. S3: The UniFrac-based hierarchical cluster analysis of all treatments. Figure. S4: The dbRDA analysis is a distance-based redundancy analysis, which is suitable for any distance matrix.
