# Supplementary material for: Influence of Biochar on Soil Nutrients and Associated Rhizobacterial Communities of Mountainous Apple Trees in Northern Loess Plateau China
Source: Microorganisms. 2022 Oct 20;10(10):2078. doi: 10.3390/microorganisms10102078 (PMC9610541; doi:10.3390/microorganisms10102078)
Supplement: Supplementary file 1 [file microorganisms-10-02078-s001.zip › microorganisms-1954381-supplementary.pdf]

Influence of biochar on soil nutrients and associated rhizobacterial communities of mountainous apple trees in northern loess plateau China

Supplementary Data

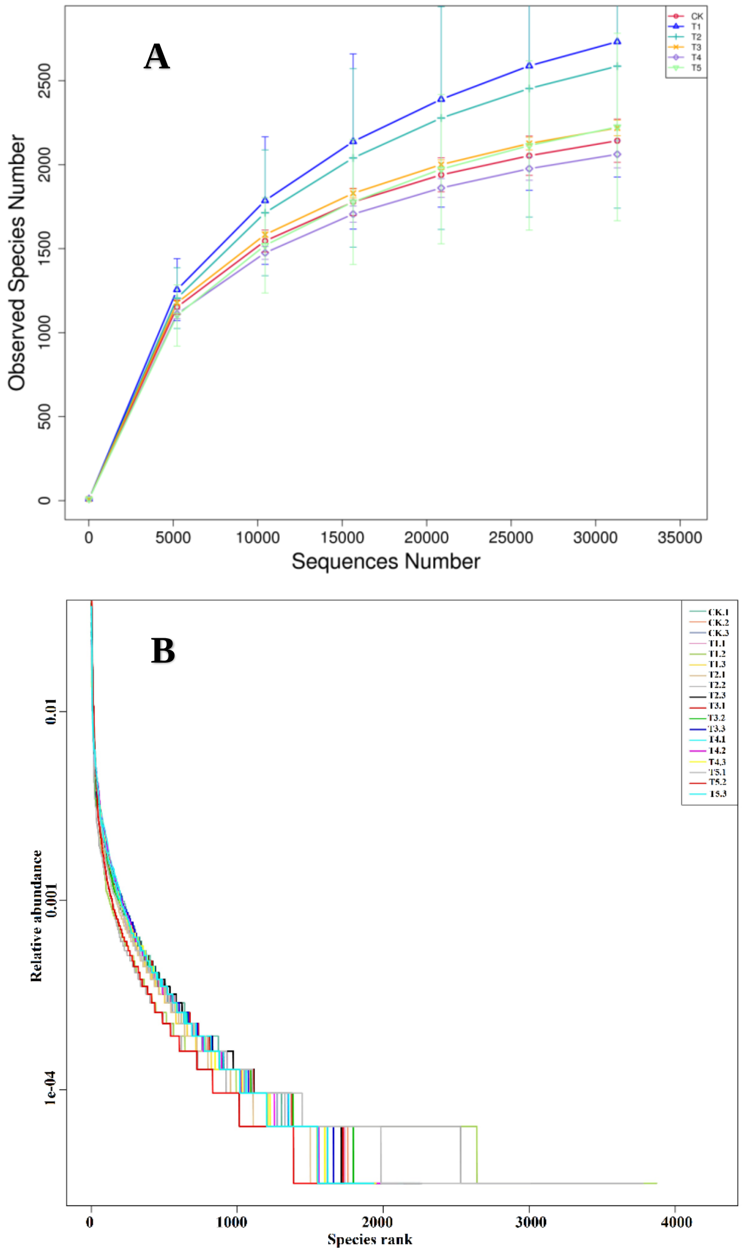

Figure S1. (A) Observed species numbers (B) Rank abundance curve in the treatments with and without biochar, different color line represents different treatments.

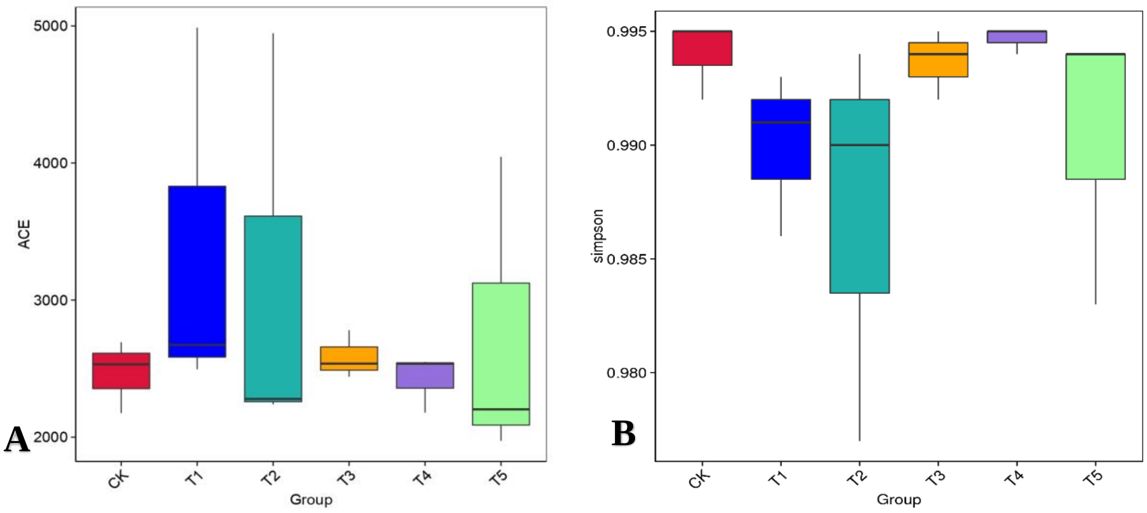

Figure S2. (A) ACE and (B) Simpson index of OTUs for soil samples where biochar was applied.

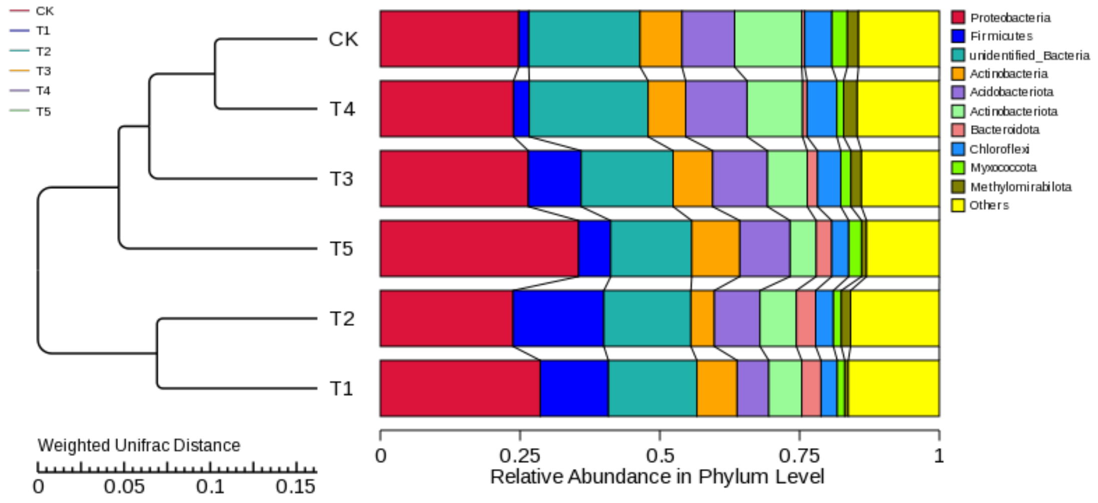

Figure S3. The UniFrac-based hierarchical cluster analysis of all treatments.

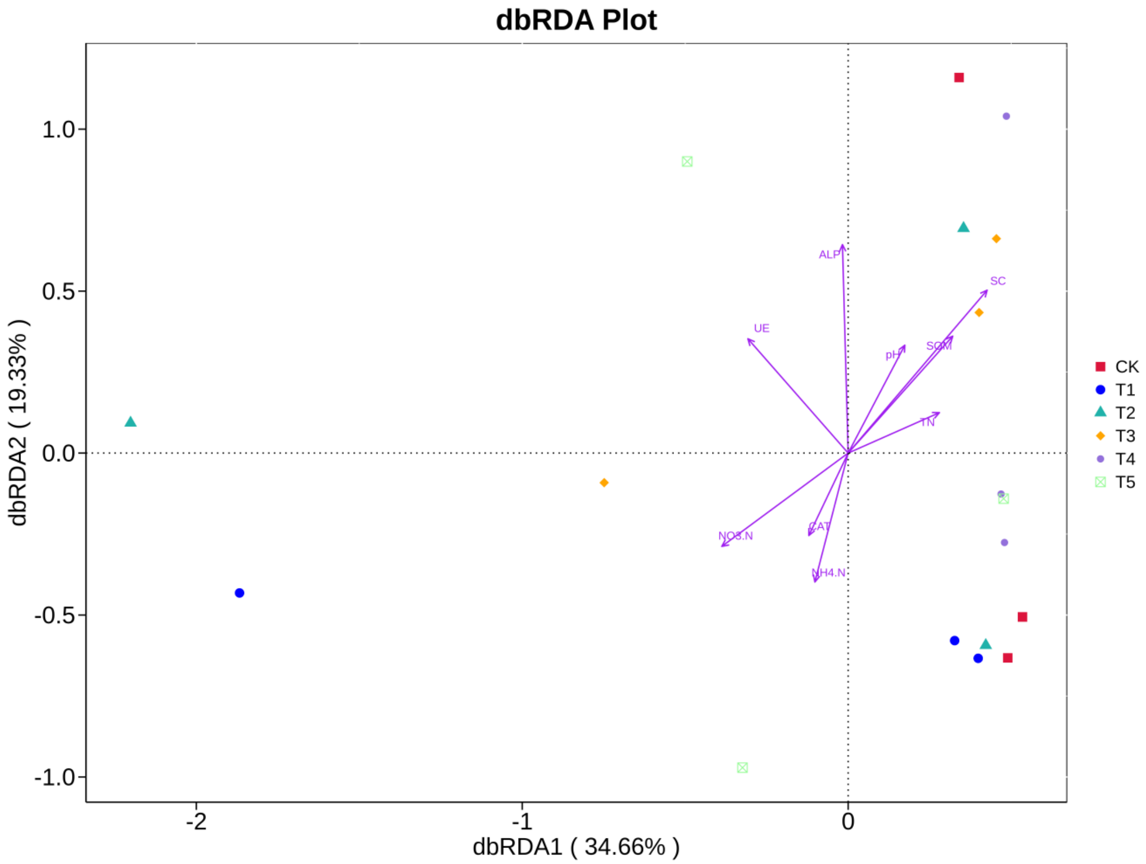

Figure S4. The dbRDA analysis is a distance-based redundancy analysis, which is suitable for any distance matrix. The calculation is based on the quantitative characteristics and environmental factors of different species in the sample.
